# Quantifying Sharpness and Nonlinearity in Neonatal Seizure Dynamics

**DOI:** 10.34133/cbsystems.0076

**Published:** 2024-01-25

**Authors:** Chien-Hung Yeh, Chuting Zhang, Wenbin Shi, Boyi Zhang, Jianping An

**Affiliations:** ^1^ School of Information and Electronics, Beijing Institute of Technology, Beijing, China.; ^2^ Key Laboratory of Brain Health Intelligent Evaluation and Intervention (Beijing Institute of Technology), Ministry of Education, Beijing, China.; ^3^ School of Engineering, University of Edinburgh, Edinburgh, UK.; ^4^ School of Cyberspace Science and Technology, Beijing Institute of Technology, Beijing, China.

## Abstract

The integration of multiple electrophysiological biomarkers is crucial for monitoring neonatal seizure dynamics. The present study aimed to characterize the temporal dynamics of neonatal seizures by analyzing intrinsic waveforms of epileptic electroencephalogram (EEG) signals. We proposed a complementary set of methods considering envelope power, focal sharpness changes, and nonlinear patterns of EEG signals of 79 neonates with seizures. Features derived from EEG signals were used as input to the machine learning classifier. All three characteristics were significantly elevated during seizure events, as agreed upon by all viewers (*P* < 0.0001). Envelope power was elevated in the entire seizure period, and the degree of nonlinearity rose at the termination of a seizure event. Epileptic sharpness effectively characterizes an entire seizure event, complementing the role of envelope power in identifying its onset. However, the degree of nonlinearity showed superior discriminability for the termination of a seizure event. The proposed computational methods for intrinsic sharp or nonlinear EEG patterns evolving during neonatal seizure could share some features with envelope power. Current findings may be helpful in developing strategies to improve neonatal seizure monitoring.

## Introduction

Neonates have a higher seizure incidence than adults [1], and full-term neonates are particularly at high risk for stroke, meningitis, and hypoxic–ischemic encephalopathy; these occurrences would require intensive monitoring in the neonatal intensive care unit (NICU). Additionally, the occurrence of neonatal seizures reflects underlying neurological dysfunction, supporting the need to characterize the epileptic patterns as well as the dynamics of neonatal seizures [2]. However, clinical reports, as subjective evaluations, might sometimes be unreliable [3–5]. For instance, Murray et al. [4] reported that all electroencephalogram (EEG) signals acquired during seizures were merely 9% in line with clinical evaluation, revealing an underestimation of the incidence rate of neonatal seizures. Such difficulty prevails in infants with subtle clinical manifestations that are rarely identified by a pure clinical performance assessment, in particular [6]. Factors that affect assessments include unsociable hours in the NICU, insufficient interrater agreement, different seizure occurrences, and the indirect relation of clinical scores to underlying neural transmission; all these factors affect the clinical scoring for widespread use.

EEG data are widely accepted by the NICU for their applicability, reliability, and accuracy and are especially useful for prolonged monitoring. Neonatal video-EEG polygraphy is a standard tool for seizure monitoring [7–9]. However, the demand to robustly screen EEG profiles is labor intensive and subjective, thus limiting mass inspections. Amplitude-integrated EEG is a popular replacement for video-EEG polygraphy due to its portability, simplified interpretation, and real-time monitoring capability [10]. It provides an overview of brain activity, enabling efficient assessment and detection of abnormal patterns. However, more evidence has shown that amplitude-integrated EEG can miss short-duration seizures [11,12], particularly segments with infrequent, focal, brief, and low-amplitude patterns [10]. For example, Rennie et al. [13] reported that cerebral function monitor traces had poor agreement among observers at all three speeds, with a sensitivity of no more than 55%.

To improve the efficiency, robustness, and reproducibility of EEG monitoring/measuring, several approaches were introduced based on EEG data either with [14] or without electrocardiogram data [15,16]. The oscillatory envelope of an EEG wave is known as an important element for distinguishing epileptiform discharges from normal EEG signals, and how these quantitative trends reflect the propagation of seizures is critical. Cotside EEG monitors provide multichannel recordings and support prolonged amplitude-integrated EEG monitoring, along with additional features such as quantitative trend analysis and video recording of movements. However, few quantitative trends can reflect waveform dynamics. The rhythmic artifacts in EEG signals may resemble those of authentic epileptic patterns, thus impeding the capture of finer waveform characteristics. To address the challenges, we propose a method that focuses on characterizing the evolution of seizures by considering the epileptic sharpness and the degree of nonlinearity in EEG signals. We aim to avoid misinterpreting the outputs cotside and infer the possible underlying neurological mechanisms. Unlike the routine approaches mainly dependent on the frequency domain, our proposed method relied on the time series per se, aiming to establish comprehensive indicators for irregular epileptic patterns with multiple morphological characteristics simultaneously considered.

In this work, we present an analysis of neonatal seizure dynamics using the proposed method. We investigate the complex envelope power, epileptic sharpness, and degree of nonlinearity in EEG signals obtained from neonates with seizures. These features serve as candidate measures to quantify the temporal dynamics of neonatal seizures. The occurrence of a seizure event is usually grounded in the consensus of clinical specialties [17,18]. However, ambiguous or insensitive situations frequently occur through visual inspections and interobserver agreement [19–21]. Considering the possible effect of the diverse judgments among viewers, we included all sets of combinations of the agreed-upon viewers, i.e., different numbers of agreed-upon viewers of the occurrence of a neonatal seizure event.

The remainder of this study is structured as follows: In Materials and Methods, we provide an overview of the materials used and describe the proposed method. In Results, we present the results of analyzing the EEG waveform in neonatal seizure dynamics. A comprehensive discussion of the findings is presented in Discussion.

## Materials and Methods

### Participants

The neonatal epileptic EEG recordings, Helsinki dataset, including a collection of EEG signals from 79 neonates with suspicious seizure events annotated, were downloaded from Zenodo [22]. Informed consent was obtained from all participants. Three inclusion criteria included (a) postmenstrual age = 35 to 45 weeks, (b) gestational age *>* 32 weeks, and (c) EEG recordings of high quality without technical artifacts (e.g., environmental noise, unwanted physiological artifacts, or improper electrode placement). All protocols were approved by the Ethics Committee of the Helsinki University Hospital, Finland (Number: HUS/2347/2016; Date: 2016 December 15). The 18 electrodes of EEG signals were positioned as per the standard 10–20 system. A reference was set at the midline. Next, the standard longitudinal bipolar layout was applied to create bipolar montages [23]. The standard criterion for visual seizure annotation introduced by Clancy et al. [24] was referenced by 3 well-trained experts. The average length of a recording was 74 min. The sampling rate of EEG traces was 256 Hz. Additional clinical details of all subjects can be found in Stevenson et al. [23].

Moreover, we incorporated the CHB-MIT dataset, collected at the Children’s Hospital Boston, which encompasses EEG recordings from pediatric subjects suffering from intractable seizures. This dataset served as a validation set for comparing our proposed methods. The EEG data were sampled at a frequency of 256 Hz and collected via 22 electrodes positioned in accordance with the international 10–20 system. This dataset encompasses roughly 1,136 h of uninterrupted EEG signal activity and encompasses 198 instances of epileptic seizure events. The ages of the patients in this dataset range from 1.5 to 22 years. Comprehensive details about the dataset can be found in Goldberger et al. [25].

### Preprocessing

First, shape-preserving piecewise cubic interpolation was applied to correct the out-of-range values. A threshold was set as an absolute value of 200. The 50-Hz tone was next removed with a second-order IIR notch filter. Then, a sixth-order Butterworth high-pass filter with a 1-Hz cutoff frequency was designed to eliminate the low-frequency contaminants from movement artifacts [25]. Additionally, all recordings were *z*-scored to minimize the effect of the impedance differences in electrodes [26]. To clarify critical information associated with neonatal seizures, principal component analysis was applied to reduce dimensionality, and at least 95% of the accumulated percentage of eigenvalues was kept with principal components arranged in descending order [27]. Of note, to avoid interrater error due to visual inspection, all types of fusion with annotations defined by multiple experts were considered, i.e., at least one, at least two, or all experts agreed with the annotation of seizure occurrence.

### Time-varying complex envelope

To compute the complex envelopes of the time-evolved EEG signals, we utilized a non-overlapping epoch with a duration of 1 s. The complex envelope was obtained using the Hilbert transform, which provides a representation of the instantaneous amplitude and phase of the signal. By taking the absolute value of the complex envelope, we extracted the magnitude component, which represents the amplitude variations of the EEG signals. This process allowed us to capture the temporal evolution of the EEG signals’ amplitudes over the 1-s intervals. Finally, the median values of these 1-s envelopes were averaged to obtain a representative value for further analysis.

### Epileptic sharpness

To assess the sharpness of epileptic oscillations, we employed a median filtering approach known as the order-statistic filter. This method allowed us to determine an adaptive threshold for detecting extreme values in EEG waves. Briefly, a sliding Tukey window was used to weigh the input with a 1-sample forward shift between realizations, and then the intersections between the weighted track and the input were defined as the output (local maxima) [28,29]. Next, the absolute voltage differences between the detected extrema and that during the 7 ms on either side were averaged, and then the median value of all averaged absolute voltage differences of one segment was defined as the epileptic sharpness of an input:

$$\begin{array}{c} \text{Sharpness}=\text{median}\\ \langle \left\{\left(\left|x_{\left(\text{peak}\right)}-x_{\left(\text{peak}-7\mathrm{ms}\right)}\right|\right.\right.{\left.\left.+\left|x_{\left(\text{peak}\right)}-x_{\left(\text{peak}+7\mathrm{ms}\right)}\right|\right)/2\right\}}_{i},i=1,2,\cdots ,N\rangle \end{array}$$ (1)

where *x*_(peak)_, *x*_(peak−7 ms)_, and *x*_(peak+7 ms)_ denote the voltages on the *i*th detected extrema and the 7 ms on either side, respectively. The total number of detected extrema within a consecutive segment is *N*. This strategy was applied to all nonoverlapping seizure segments. The detailed procedure to assess the sharpness of an oscillation can also be found in the literature [28,30].

### Degree of nonlinearity

The degree of nonlinearity (DoN), unlike the sharpness of an oscillation, estimates the degree of waveform distortions based on the consecutive instantaneous frequency (IF). Therefore, all sample points of an evolving time series mattered. We implemented the definition by Huang et al. [31] in estimating the nonlinearities of an irregular EEG signal:

$${\mathrm{DoN}}_{i}=\mathrm{std}\langle \frac{{\left(\text{IF}\right)}_{i}-{\left({\text{IF}}_{z}\right)}_{i}}{{\left({\text{IF}}_{z}\right)}_{i}}\rangle$$ (2)

where *IF* and *IF_z_* represent the instantaneous and zero-crossing frequencies, respectively. Notably, *IF* = d*θ*(*t*)*/*d*t*, where *θ*(*t*) stands for the Hilbert phase function. To provide insight into the intrawave frequency modulations, *DoN_i_* is defined as the standard deviation of normalized instantaneous frequencies (intrawave modulation) referenced to that of the zero-crossing frequencies (interwave modulation) for the *i*th segment. This measure has been implemented in previous studies to characterize physiological and/or pathophysiological oscillations [28,32]. In this work, DoN, along with epileptic sharpness and the complex envelope, was derived from neonatal EEG recordings. Both the seizure and the nonseizure periods were estimated separately, of which the former was further segmented into the starting, middle, and end seizure stages in equal length.

### Statistical analysis

A statistical analysis aimed to test the ability of the complex envelope, sharpness, and DoN biomarkers in distinguishing the neonatal seizure periods/stages, locations of EEG electrodes, and the number of agreed-upon viewers. Repeated analysis of variance (ANOVA) was applied, with subjects considered as a random factor. A 2-tailed test with *P* value less than 0.05 was considered to be statistically significant (*α* = 0.05 for all hypothesis testing). Apart from *P* value, *F* value and degree of freedom were provided to compare probability distributions and ensure an appropriate number of unrestricted and independent variables per factor. Tukey’s honest significance test, as a correction for multiple comparisons, was used to test all possible pairwise differences of means. Variables were box-cox transformed to adapt potential non-normal dependent variables into a normal shape for significance analyses. Similar statistical approaches were applied to perform the tests under all fusions of multiple expert annotations. The variables in all tables were categorized as follows: “period” included 2 categories, indicating seizure or nonseizure; “contact” comprised 18 bipolar electrode contact pairs; and “stage” encompassed the 3 stages of seizure progression, namely, the starting, middle, and end seizure stages.

### Classification of features

The classification process involved the examination of 3 features: complex envelope power, epileptic sharpness, and DoN. To assess the diagnostic performance of these features for neonatal seizure estimation, receiver operating characteristic (ROC) curves were used. To validate the model, 20% of the data were reserved for testing, while the remainder underwent training via 5-fold cross-validation. For classification, support vector machine (SVM), k-nearest neighbors (kNN), logistic regression (LR), and Naive Bayes (NB) were employed. To ensure the reliability of the model, the agreement of seizure events among all viewers was established. Additionally, the calibration curves of the proposed features were utilized to assess the model’s reliability. This assessment involved comparing the outcomes predicted by the features with those determined by clinical experts, providing insight into the accuracy and consistency of the classification results. Notably, in our analysis of the classification performance of the 3 features across all channels, we observed that globally, C4-P4 demonstrated the highest performance in terms of area under the curve (AUC). As a result, we demonstrated the classification performance of oscillatory features specifically on C4-P4.

## Results

### Complex envelopes

**Fig. 1.**
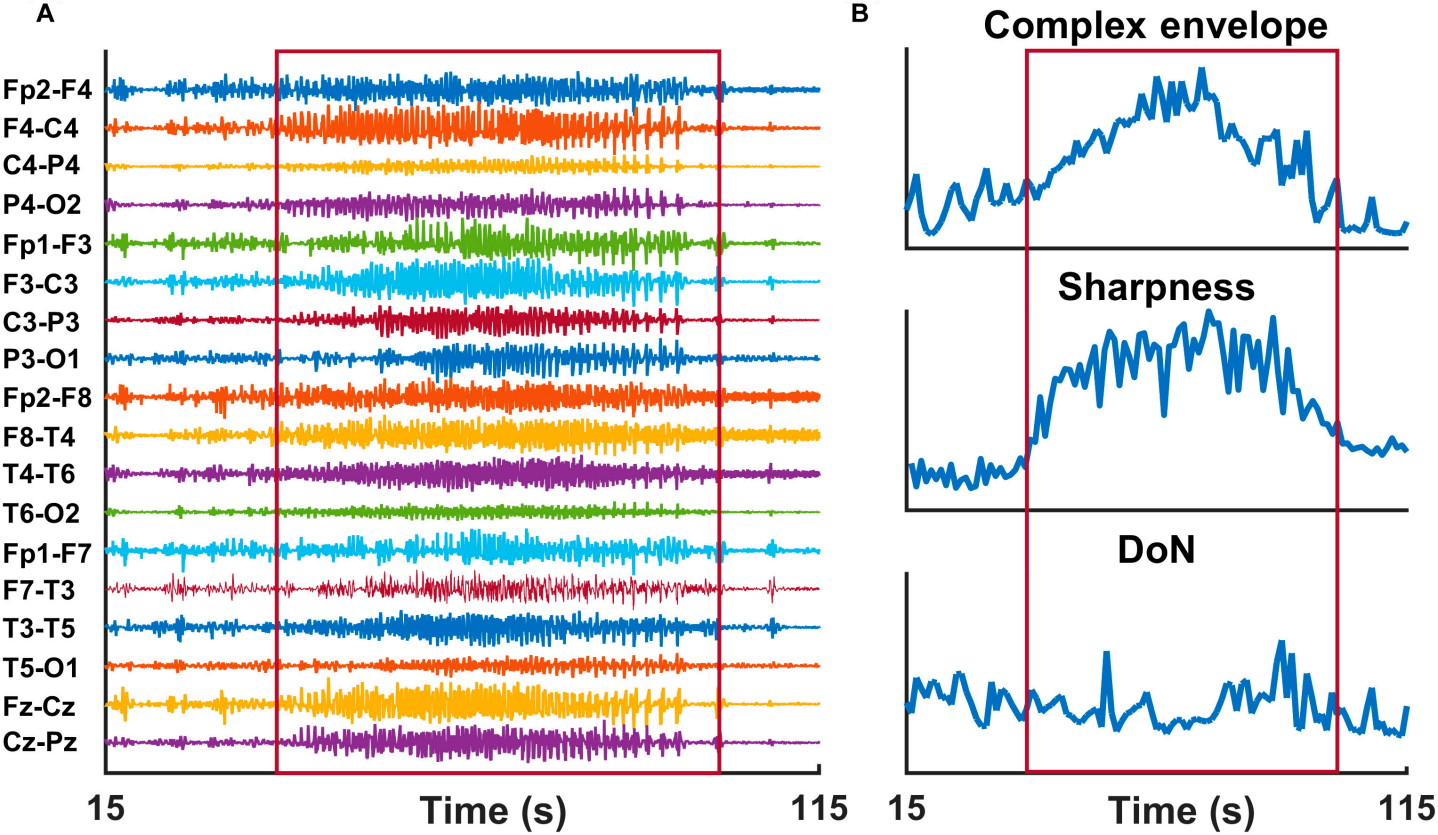
A demonstration of (A) multi-channel neonatal preprocessed EEGs, and (B) the corresponding tracks of the complex envelope, sharpness, and DoN. The red rectangles indicate the seizure periods.

**Fig. 2.**
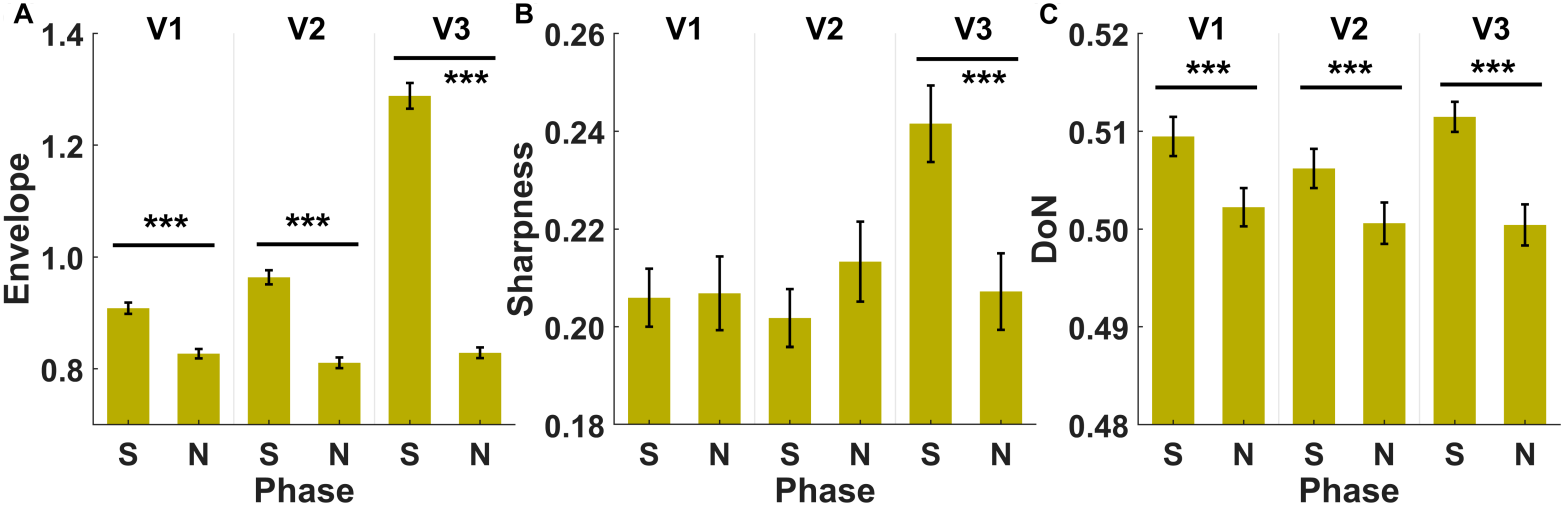
Comparisons of periods (seizure and nonseizure) using neonatal epileptic EEG characteristics include (A) the complex envelope, (B) epileptic sharpness, and (C) DoN. All comparisons are grouped by the number of the agreed-upon viewers (1, 2, and 3). ***P < 0.0001.

Figure 1A demonstrates a typical example of multichannel (18 bipolar electrodes) preprocessed neonatal EEG signals. The red rectangles indicate the annotated occurrence of seizures. As shown in the uppermost panel of Fig. 1B, there is a trend of a rising complex envelope during the seizure periods compared to the nonseizure periods nearby. We began by testing whether the complex envelope was modulated by seizure events, electrode contact pairs, and/or the number of agreed-upon viewers. The ANOVA data summarized in Table S1 demonstrates that there are significant effects on the seizure events (*P* < 0.0001), the number of agreed-upon viewers (*P* < 0.0001), and the electrode contact pairs (*P* < 0.0001). Considering a significant interaction between the seizure events and the number of agreed-upon viewers (*P* < 0.0001), the post hoc test results shown in Table S2 indicate that the complex envelope is greater during a seizure than during nonseizure periods with all numbers of agreed-upon viewers (*P* < 0.0001); this outcome is especially prominent when the seizure events were determined by the consensus of all viewers (see the rightmost panel in Fig. 2A). Of note, the complex envelope during the nonseizure periods was approximately the same for all numbers of agreed-upon viewers, whereas the envelope during the seizure periods was higher in the condition with more agreed-upon viewers, as shown in Fig. 2A.

Considering that a complex envelope corresponds to the integration of power across multiple frequency bands, we explored each band power of clinical interest to link an individual to the whole. Spectral analyses were performed on the 1-s nonoverlapping epochs multiplied by a Hamming window, followed by averaging all 1-s band powers. The frequency bands of interest include delta (1 to 4 Hz), theta (4 to 9 Hz), alpha (9 to 13 Hz), beta (13 to 30 Hz), and gamma (>30 Hz) bands. During a seizure, in general, delta band power rose, and the higher-frequency power (alpha, beta, and gamma bands) decreased, as expected. Our results reveal that the delta power was greater for seizure periods than for nonseizure periods (Fig. S1A), while the high-frequency power (i.e., alpha, beta, and gamma bands, which correspond to Fig. S1C to E) all reached a significant level (*P* < 0.0001), except for the theta power, regardless of the number of agreed-upon viewers (Fig. S1B). A significant difference only occurred when one viewer agreed on the seizure event.

Delta power is the only band power higher for a seizure event, which validates past findings that the complex envelope mainly reflects the delta power for neonatal seizures [33]. The generally strong significant differences in envelope power (*P* < 0.0001) across all numbers of agreed-upon viewers (Fig. 2A) compared to that of individual band power, shown in Fig. S1, imply that the complex envelope is a highly reliable indicator for identifying seizure events. Such a method (complex envelope) eliminates the use of a window function in constraint temporal resolution, all supporting the complex envelope as an ideal use in the following analyses.

**Fig. 3.**
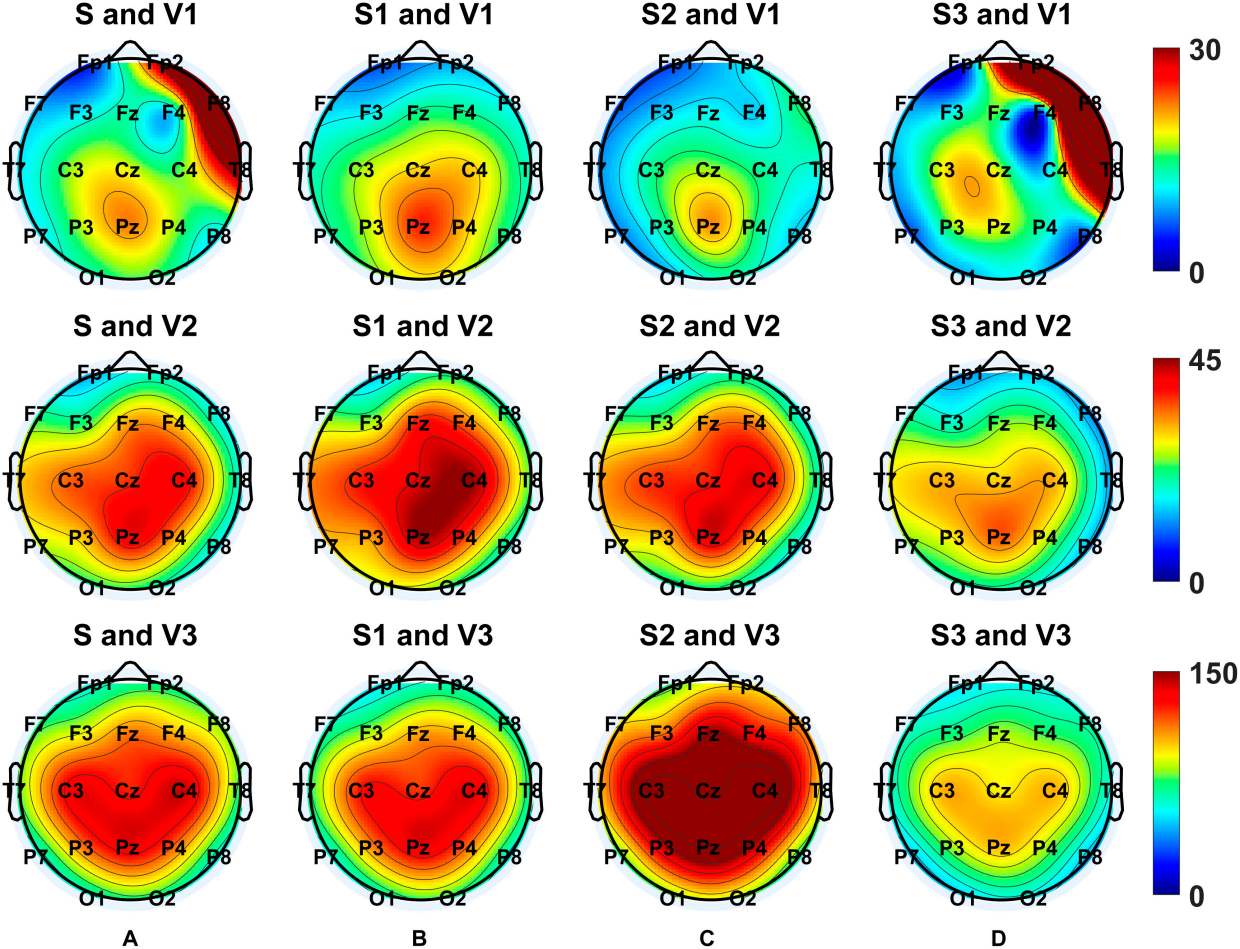
Brain maps of complex envelope power under the (A) whole (S), (B) starting (S1), (C) middle (S2), and (D) end (S3) stages of seizure relative to the nonseizure period. V1, V2, and V3 correspond to the number of the agreed-upon viewers as 1, 2, and 3, respectively.

Therefore, we further examined the topological changes in envelope power during seizure periods relative to nonseizure periods across the whole brain. The brain maps in Fig. 3A show the relative envelope power (%) in conditions with different numbers of agreed-upon viewers (top-down topography plots: V1, V2, and V3). The strongest relative envelope power was revealed around Cz-Pz, with a gradient decreasing to the outer-ring brain maps, while the seizure events were determined by the consensus of 2 viewers or more. It is worth noting that the envelope power in the condition with 3 viewers tended to be the most symmetrically distributed one.

### Epileptic sharpness

The waveforms of EEG signals during the seizure periods seem to become sharper (see the red rectangle in the middle panel of Fig. 1B). To confirm this point, we tested whether sharpness was modulated by seizure events, the electrode contact pairs, and/or the number of agreed-upon viewers. The ANOVA data summarized in Table S1 show that there is a significant effect on the seizure events (*P* < 0.0001), the electrode contact pairs (*P* < 0.0001), and the number of agreed-upon viewers (*P* = 0.0089). Meanwhile, significant interactions were found in seizure events with both the electrode contact pairs (*P* = 0.0003) and the number of agreed-upon viewers (*P* < 0.0001). Next, the post hoc test results shown in Table S2 further reveal that sharpness is significantly greater during seizure periods than during nonseizure periods if the annotations are given based on the consensus of all viewers (*P* < 0.0001) (see the rightmost panel in Fig. 2B). The trends are opposite for the rest of the conditions (see the left 2 panels in Fig. 2B). Our results indicate that the characteristics of sharpness may differ between recognized and nonevident seizure events (i.e., ambiguous seizure events have less epileptic sharpness).

**Fig. 4.**
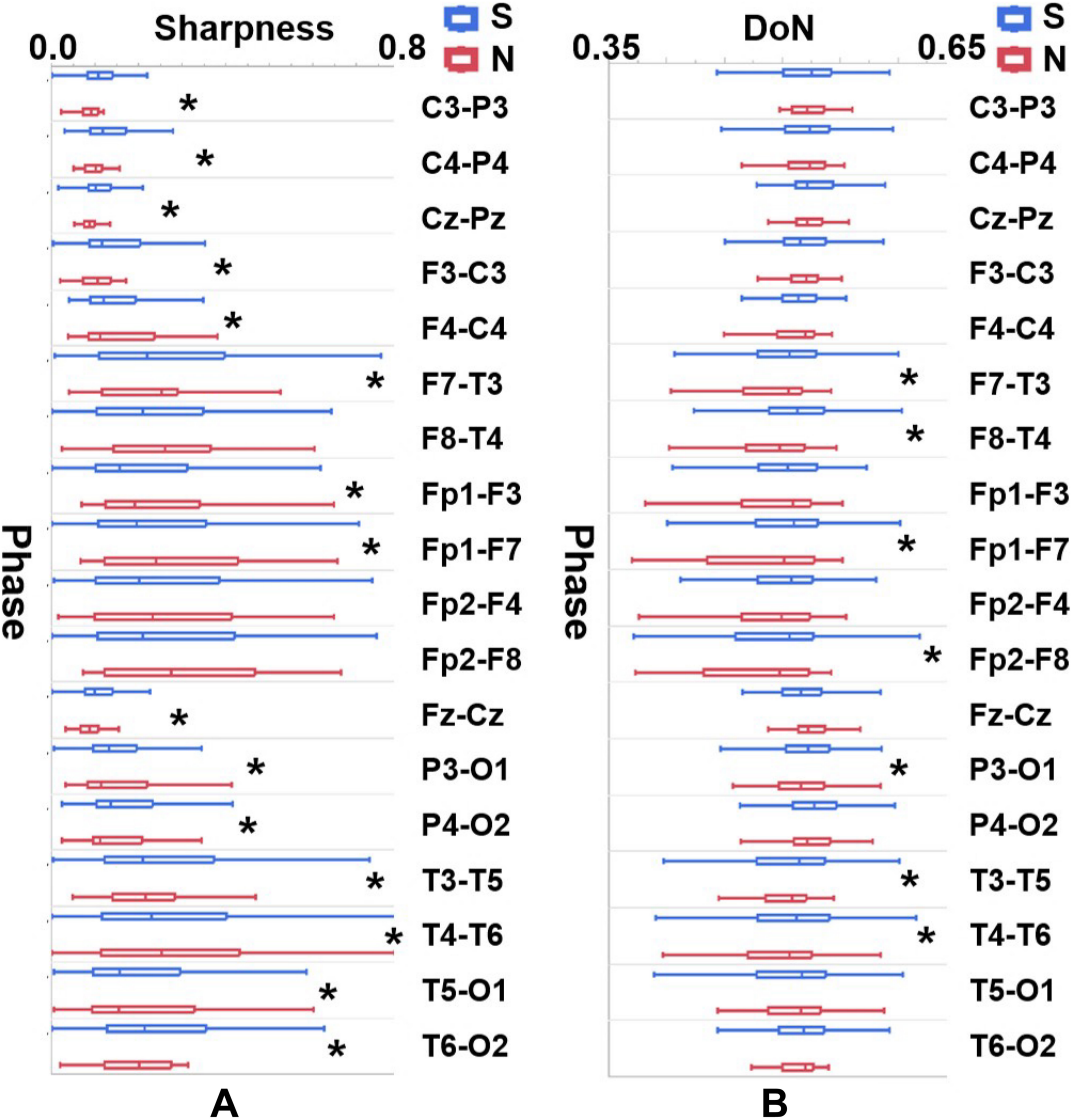
Comparisons of periods (seizure and nonseizure) using neonatal epileptic EEG characteristics include (A) epileptic sharpness and (B) DoN. All comparisons are grouped by the electrode contact pairs. *P < 0.05.

**Fig. 5.**
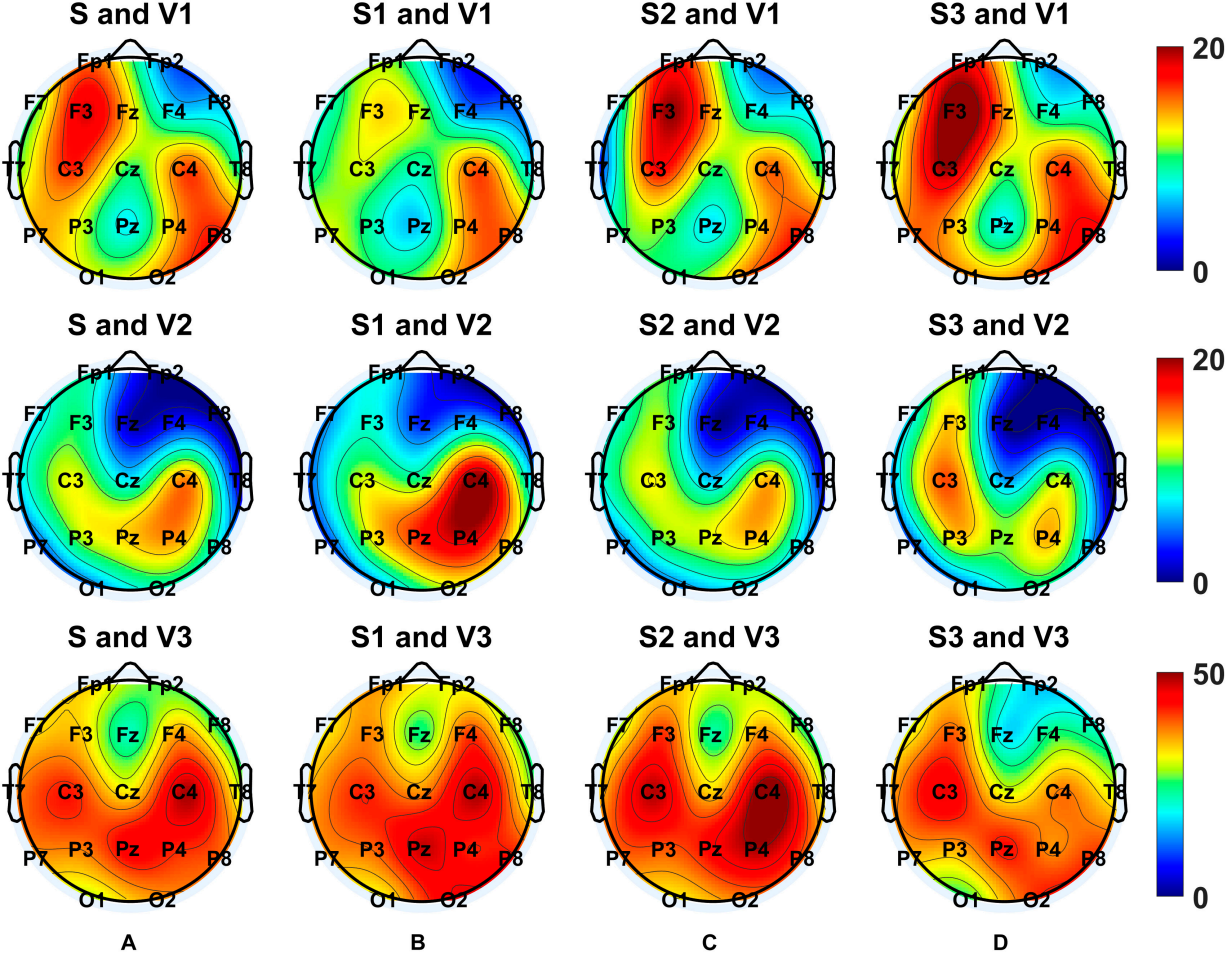
Brain maps of epileptic sharpness under the (A) whole (S), (B) starting (S1), (C) middle (S2), and (D) end (S3) stages of seizure relative to the nonseizure period. V1, V2, and V3 correspond to the number of the agreed-upon viewers as 1, 2, and 3, respectively.

We next examined whether sharpness was modulated by seizure events and the number of agreed-upon viewers, grouped by the 18 electrode contact pairs. Table S3 shows that 15 contact pairs presented significant differences in sharpness between seizure and nonseizure periods, except in the right frontal brain area (Fp2-F4, Fp2-F8, and F8-T4). Among these, 10 contact pairs had significantly sharper waveforms during seizure periods than during nonseizure periods (Fig. 4A). The brain maps in Fig. 5A show the relative sharpness during the seizure periods relative to nonseizure periods (top-down topography plots: V1, V2, and V3). When the seizure periods were determined by the consensus of all viewers, stronger relative sharpness was revealed around C3-Pz-C4 with a gradient decrease toward Fz. Importantly, the brain maps with fewer agreed-upon viewers tended to be less symmetrically distributed, especially for V1 (see the uppermost panel in Fig. 5A).

### Characteristics of nonlinearity

The waveforms of EEG signals seem to become more irregular toward the end of a seizure segment (see the red rectangle in the lowest panel of Fig. 1B). It should be noted that the preprocessed EEG recordings were carefully examined to guarantee zero-crossing patterns with a fair estimation of instantaneous frequencies. Therefore, we last explored whether the DoN was modulated by the seizure events, the electrode contact pairs, and/or the number of the agreed-upon viewers of the seizure events. The ANOVA data summarized in Table S1 demonstrate that there is a significant effect on the seizure events (*P* < 0.0001), as well as the electrode contact pairs (*P* < 0.0001). Meanwhile, a significant interaction was shown between the seizure events and the electrode contact pairs (*P* = 0.0249). We compared the DoN during the seizure periods to that during nonseizure periods with a different number of agreed-upon viewers. As shown in Fig. 2C, all tests revealed significantly higher DoNs in the seizure periods than in the nonseizure periods (*P* < 0.0001), as expected.

**Fig. 6.**
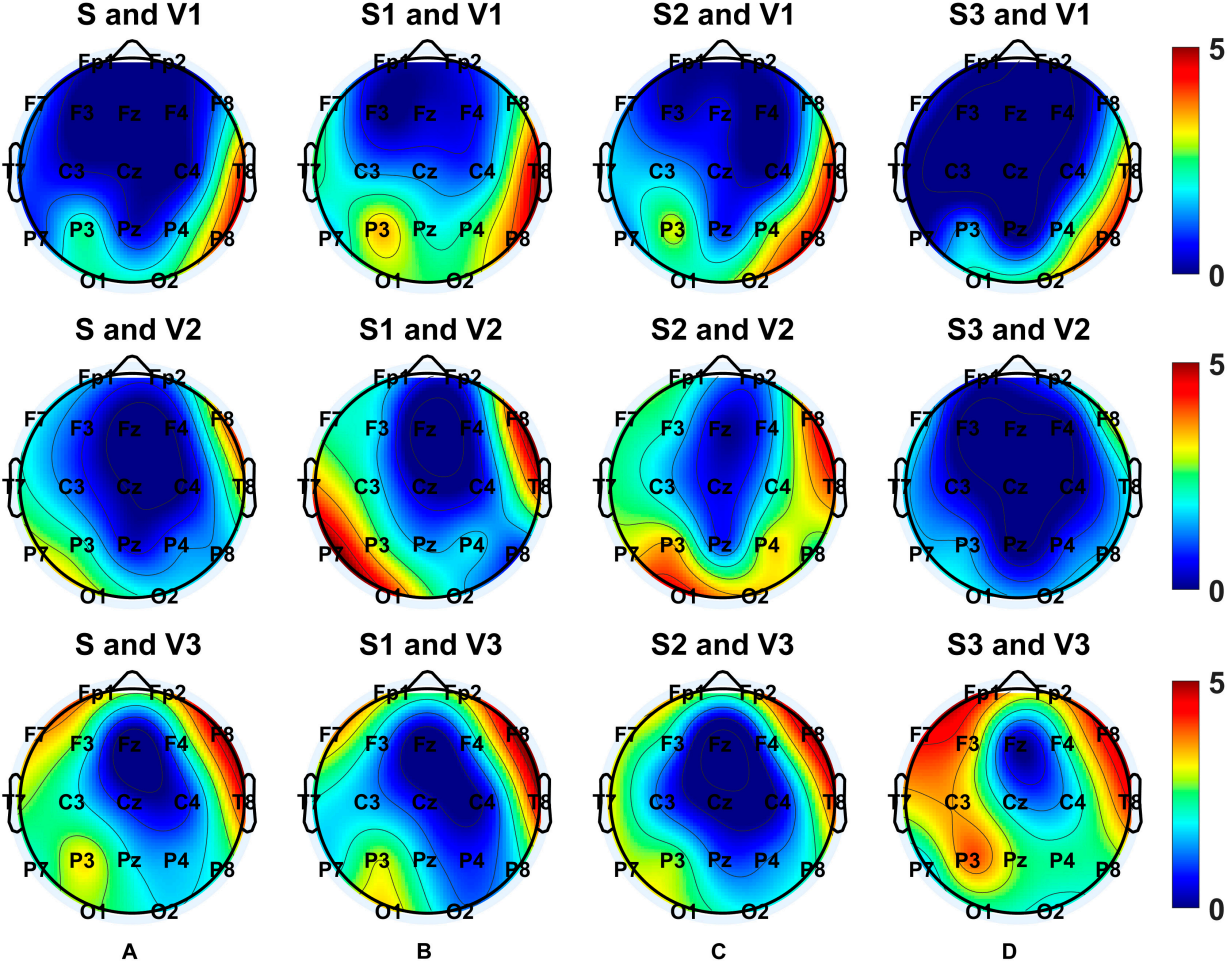
Brain maps of DoN under the (A) whole (S), (B) starting (S1), (C) middle (S2), and (D) end (S3) stages of seizure relative to the nonseizure period. V1, V2, and V3 correspond to the number of the agreed-upon viewers as 1, 2, and 3, respectively.

Similarly, we also explored whether DoN was modulated by seizure events and the number of agreed-upon viewers on seizure events, grouped by the 18 electrode contact pairs. Table S3 shows that 7 of 18 contact pairs, basically those surrounding the peripheral region of the brain map, had significant differences in DoN between the seizure and the nonseizure periods. Among these contacts, all 7 contact pairs presented significantly higher DoNs in the seizure periods than in the nonseizure periods (Fig. 4B). In Fig. 6A, all topography plots (top-down topography plots: V1, V2, and V3) show fewer differences between the seizure and the nonseizure periods for DoNs around Fz, with V3 presenting the highest kurtosis in the DoN distribution topography (see the lowermost panel).

### Progression of epileptic patterns

**Fig. 7.**
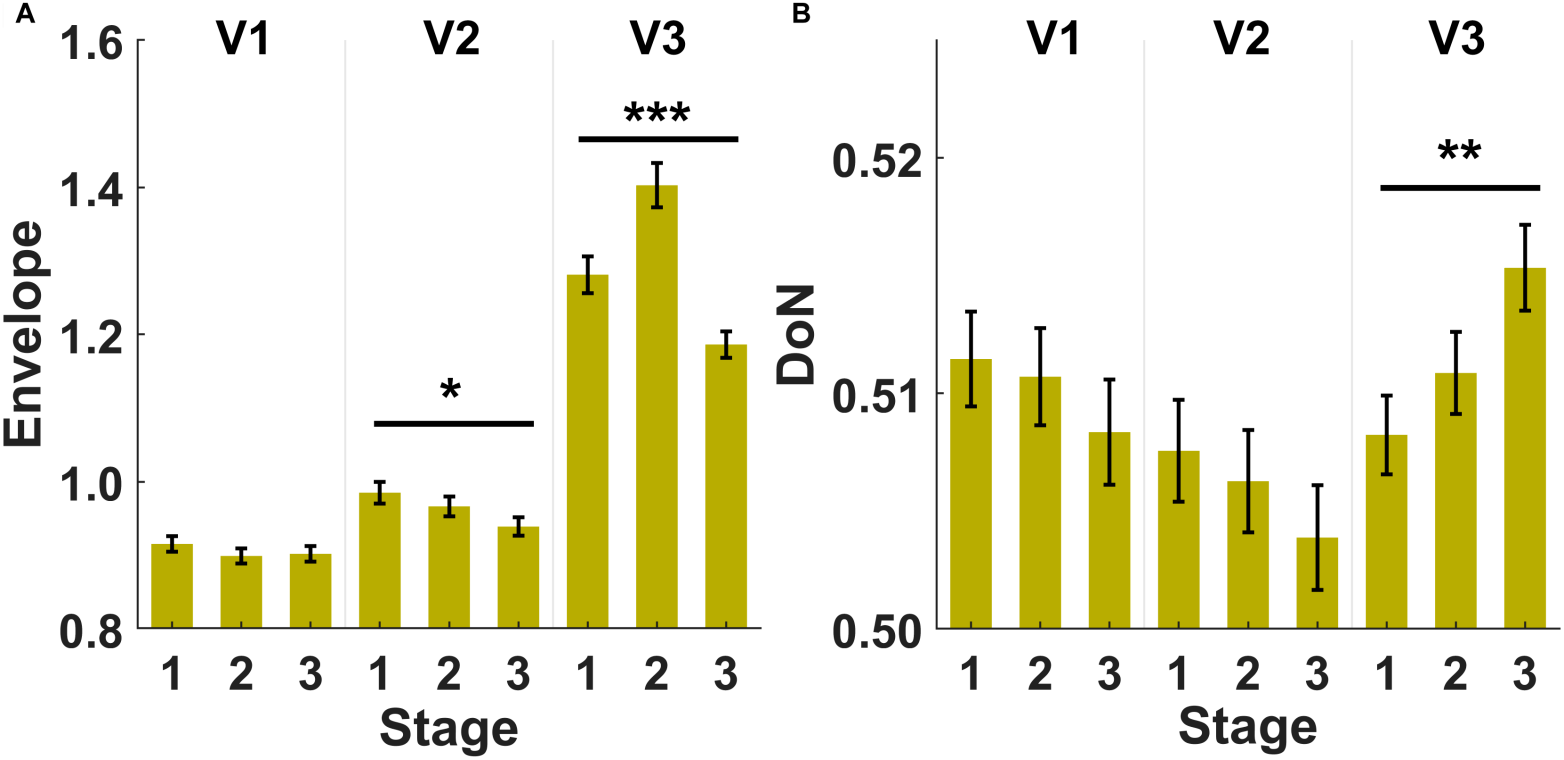
Comparisons of stages (starting, middle, and end seizure stages), grouped by the number of the agreed viewers (1, 2, and 3), using neonatal epileptic EEG characteristics including (A) complex envelope and (B) DoN. *P < 0.05, **P < 0.001, and ***P < 0.0001.

Next, we explored whether the shape of the epileptic pattern and its envelope power progressed within the seizure periods. We hypothesized that nonlinearity might increase to terminate epileptic patterns along with misaligned neural activities. To this end, each seizure event was further subsegmented into 3 equal-length sequences (i.e., the starting, middle, and end stages), of which characteristics, including complex envelope, sharpness, and DoN, were estimated in each sequence. In Table S4, the results of an ANOVA of the complex envelope are shown, and all factors, including subsegments, electrode contact pairs, and the number of agreed-upon viewers of seizure events, all revealed significant effects (*P* < 0.0001). The latter was found to have significant interactions with both subsegment (*P* < 0.0001) and electrode contact pairs (*P* = 0.0038). Next, post hoc tests in Table S5 indicated that there is a significantly highest complex envelope in the middle stage (stage 2) followed by the starting (stage 1) and then the end (stage 3) stage with the consensus of all viewers (*P* < 0.0001; see the rightmost panel in Fig. 7A). However, stage 1 tends to be the highest stage with V2 (*P* = 0.0118; see the middle panel in Fig. 7A). For the topography plots (top-down topography plots: V1, V2, and V3) of the envelope power during finer segmentation within a seizure event relative to the nonseizure periods (stages 1 to 3 correspond to Fig. 3B to D, respectively), the brain maps with V3 tend to be the most symmetrically distributed set. Among them, the contact pair Cz-Pz presents the highest complex envelope. However, the relative envelope power dynamics with V2 and V3 fail to be consistent.

Similarly, an ANOVA of sharpness with factors including subsegments, electrode contact pairs, and the number of agreed viewers on seizure events was examined, revealing significant effects on the latter 2 factors (*P* < 0.0001). No significant interactions were shown (Table S4). In general, the topography plots (top-down topography plots: V1, V2, and V3) of sharpness during a finer seizure stage (1 to 3 correspond to Fig. 5B to D, respectively) relative to the nonseizure periods revealed less relative sharpness (%) at Fz, while sharpness was greater around C3 and C4. These differences fail to reach a significant level.

Finally, as shown in Table S4, an ANOVA of DoN with factors including subsegments, electrode contact pairs, and the number of agreed-upon viewers of seizure events was also examined. Significant effects were shown on electrode contact pairs (*P* < 0.0001), the number of agreed-upon viewers (*P* = 0.0370), and an interaction between the subsegments and the number of agreed-upon viewers (*P* = 0.0299). The post hoc test results in Table S5 indicate that there is a significantly highest DoN in the end stage (stage 3) followed by the middle stage (stage 2) then the starting (stage 1) stage with the consensus of all viewers (*P* = 0.0023; see the rightmost panel in Fig. 6B). The topography plots (top-down topography plots: V1, V2, and V3) of the DoN during a finer seizure stage (1 to 3 correspond to Fig. 6B to D, respectively) relative to the nonseizure periods show lower relative DoN (%) around Fz. Meanwhile, the end stage with V3 (the lowermost panel in Fig. 6D) tends to have the most focalized distribution.

### Comparative performance analysis

To validate the effectiveness of our proposed method, we compared it with two contemporary methods widely employed in similar contexts: energy ratio (ER) and root mean square (RMS). ER is a conventional metric utilized to quantify the proportion of high-frequency band power (beta and gamma) relative to the low-frequency component (theta and alpha). On the other hand, RMS provides a measure of the overall magnitude of a signal, irrespective of its frequency content.

Firstly, we examined whether ER and RMS were modulated in response to the seizure event and its progression within the seizure periods for the Helsinki dataset. Generally, during a seizure event, ER demonstrated a decrease, while RMS displayed an increase, aligning with the expected behavior of band power. Our findings indicate that ER is greater during nonseizure periods compared to seizure periods (Fig. S2A) with more than one agreed-upon viewer, whereas RMS consistently attains statistical significance (*P* < 0.0001) regardless of the number of agreed-upon viewers (Fig. S2B). As depicted in Fig. S3A, no significant interactions were observed in ER, indicating its limitations in capturing the progression within the seizure periods. Conversely, there is a significantly highest RMS in the middle stage (stage 2) followed by the starting (stage 1) and then the end (stage 3) stage with the consensus of all viewers (*P* < 0.0001; see the rightmost panel in Fig. S3B). However, stage 1 predominantly displayed the highest stage with V2 (*P* = 0.0179; see the middle panel in Fig. S3B), aligning with the dynamics of envelope power.

Next, we applied all the features to the CHB-MIT dataset and examined whether their performances were consistent between the 2 datasets. As depicted in Fig. S4, complex envelope power, sharpness, RMS, and gamma power showed expected performances, with significant differences between seizure and non-seizure periods (*P* < 0.0001). However, delta and theta power showed an opposite trend between the 2 datasets. DoN exhibited a comparable level between seizure and nonseizure periods, possibly affected by the older age group for the CHB-MIT dataset. Furthermore, we utilized complex envelope power, DoN, ER, and RMS in this dataset to assess their performances in reflecting seizure progression. Similarly, the values of envelope and RMS were significantly the highest in the middle stage, but unlike the Helsinki dataset, the order of the end and the starting stages was opposite. Notably, the post hoc test indicated that the envelope performed better in distinguishing the middle from end stages (Fig. S5A), while RMS failed to differentiate between these two stages (Fig. S5D). Similar to the Helsinki dataset, DoN also demonstrated a promising performance in highlighting the termination of a seizure, exhibiting significantly higher values in the end stage (stage 3) than the others (Fig. S5B), while ER once again confirmed its inability to reflect the progression of seizures (Fig. S5C).

In summary, the performance of complex envelope, sharpness, and DoN is consistent across the 2 datasets, demonstrating the superiority and reliability of our method in identifying epilepsy and its developmental stages.

### Feature classification and diagnostic performance

Finally, we employed 3 features, namely, complex envelope power, epileptic sharpness, and DoN, to classify the diagnostic performance of neonatal seizures. Table displays the results derived from all examined classifiers utilizing these distinct features. Upon comparing the best accuracy and AUC values in Table, it is evident that for the first 2 classification schemes (seizure vs. nonseizure period, starting stage vs. nonseizure period), SVM outperformed the other classifiers, achieving accuracies of 80.0%, 91.7%, 61.5%, 93.3%, 91.7%, and 61.5%, respectively. Concerning the performances of the end stage (stage 3) referenced to that of the starting stage (stage 1), LR, kNN, and SVM achieved the best accuracy using 3 different features, respectively. Therefore, we used SVM as the classifier for further analyses.

**Table.** Performances of the tested classifiers in complex envelope power, epileptic sharpness, and DoN. Within each classification, bold fonts denote the highest accuracy and AUC values. In cases where accuracies are equal, AUC values were compared to determine the superior performance.

Envelope					Sharpness					DoN				
Classifier	Acc	Sens	Spec	AUC	Classifier	Acc	Sens	Spec	AUC	Classifier	Acc	Sens	Spec	AUC
Seizure vs. nonseizure														
SVM	**80.0%**	0.75	0.86	**0.88**	SVM	**91.7%**	1.00	0.83	**0.94**	SVM	**61.5%**	0.86	0.33	0.57
kNN	70.6%	0.88	0.56	0.72	kNN	70.6%	0.50	0.89	0.70	kNN	57.1%	0.50	0.67	**0.58**
LR	80.0%	0.75	0.86	0.73	LR	91.7%	1.00	0.83	0.86	LR	46.2%	0.71	0.17	0.36
NB	80.0%	0.62	1.00	0.79	NB	83.3%	0.83	0.83	0.89	NB	53.8%	1.00	0.00	0.55
Stage 1 vs. nonseizure														
SVM	**93.3%**	0.88	1.00	**0.98**	SVM	**91.7%**	0.86	1.00	**0.94**	SVM	61.5%	0.71	0.50	0.43
kNN	56.3%	0.67	0.43	0.55	kNN	58.3%	1.00	0.00	0.50	kNN	**61.5%**	1.00	0.00	**0.57**
LR	86.7%	0.88	0.88	0.98	LR	83.3%	0.86	0.80	0.94	LR	53.8%	1.00	0.00	0.57
NB	86.7%	0.75	1.00	0.95	NB	83.3%	0.86	0.80	0.94	NB	53.8%	0.71	0.33	0.57
Stage 1 vs. stage 3														
SVM	46.7%	0.14	0.75	0.62	SVM	57.1%	0.57	0.57	0.61	SVM	**73.3%**	0.75	0.71	0.71
kNN	56.3%	0.83	0.4	**0.68**	kNN	**64.3%**	0.80	0.56	**0.68**	kNN	68.8%	0.83	0.60	0.72
LR	**66.7%**	0.57	0.75	0.62	LR	35.7%	0.14	0.57	0.27	LR	66.7%	0.50	0.86	**0.75**
NB	53.3%	0.29	0.75	0.59	NB	42.9%	0.71	0.14	0.29	NB	66.7%	0.75	0.57	0.75

**Fig. 8.**
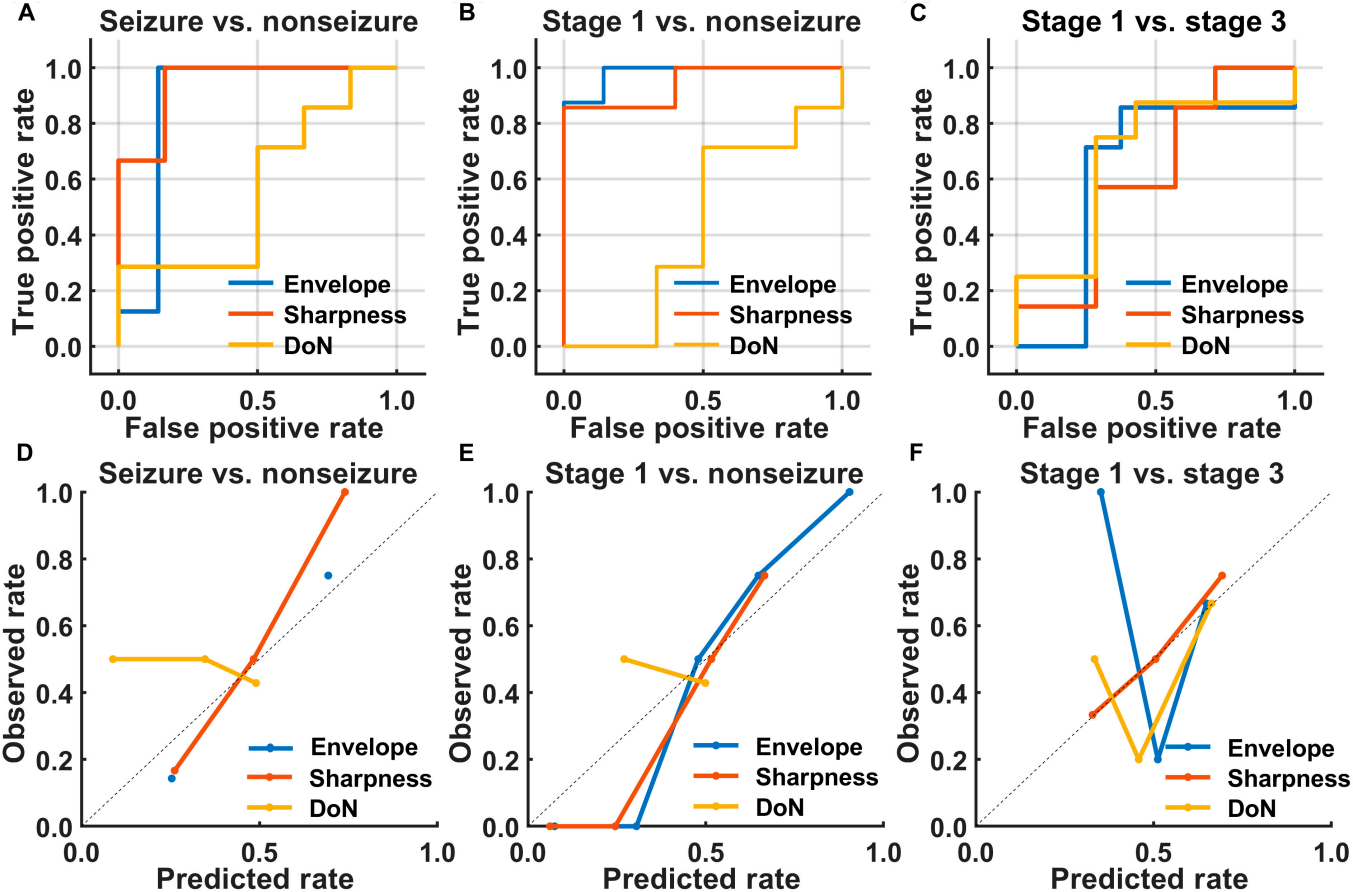
ROC curves and calibration curves for neonatal seizure feature screening. The 3 features include complex envelope power, epileptic sharpness, and DoN. The 3 classification schemes include (A and D) seizure vs. nonseizure period, (B and E) starting stage vs. nonseizure period, and (C and F) end stage vs. starting stage.

To distinguish neonatal seizures, we assessed the diagnostic performances of seizure and nonseizure periods. Of the 3 features used in SVM, sharpness demonstrated the highest accuracy and AUC. It exhibited a sensitivity of 1.00 and a specificity of 0.83. Figure 8A shows that AUCs in classifying the seizure and the nonseizure periods were 0.88, 0.94, and 0.57, corresponding to complex envelope power, epileptic sharpness, and DoN, respectively. All electrode contact pairs had AUCs higher than 0.6 with complex envelope power; meanwhile, almost all pairs had such AUCs for sharpness (15 out of 18) and DoN (17 out of 18). In Fig. 8D, the calibration curve of sharpness shows good agreement between the predicted and observed rates for seizure occurrence. The envelope power exhibited similar reliability with both low and high predicted rates (i.e., at 30% or 70%).

To monitor the evolution of a neonatal seizure, we estimated the diagnostic performances of the starting stage (stage 1) with the nonseizure period using complex envelope power, epileptic sharpness, and DoN. Among these, complex envelope power outperformed the other 2 features in terms of accuracy and AUC. The specificity was 0.88, and the sensitivity was 1.00. Figure 8B shows that the AUCs were 0.98, 0.94, and 0.43 for complex envelope power, epileptic sharpness, and DoN, respectively. For complex envelope power, sharpness, and DoN, 17, 14, and 16 electrode contact pairs respectively displayed AUCs exceeding 0.6. Figure 8E shows that both complex envelope power and sharpness had good agreement between the predicted and the observed rates for the beginning stage of the seizure (i.e., stage 1 vs. nonseizure) when a neonate is at a higher possibility of experiencing a seizure event. Meanwhile, DoN may serve as an auxiliary judgment feature when the observed possibility is close to 50%.

Likewise, we estimated the neonatal seizure diagnostic performances of the end stage (stage 3) referenced to that of the starting stage (stage 1) using complex envelope power, epileptic sharpness, and DoN. In the case of DoN, we obtained a sensitivity of 0.75 and a specificity of 0.71. Figure 8C shows that the AUCs were 0.62, 0.61, and 0.71 for complex envelope power, epileptic sharpness, and DoN, respectively. About one-third (11 out of 18) of the electrode contact pairs had AUCs higher than 0.6 with complex envelope power, and approximately half of the pairs were for sharpness (8 out of 18) and DoN (9 out of 18). According to Fig. 8F, epileptic sharpness showed higher reliability than the remaining two features in predicting the termination of a seizure event (stage 3), whereas DoN and complex envelope power may serve as supplementary judgment features when the observed likelihood exceeds 60%.

**Fig. 9.**
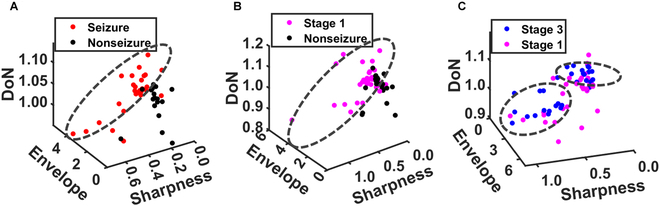
Scatter plots represent the distribution of the points for the 3 features (complex envelope power, epileptic sharpness, and DoN). The 3 classification schemes include (A) seizure vs. nonseizure period, (B) starting stage vs. nonseizure period, and (C) end stage vs. starting stage.

Finally, we explored whether combining the merits of multiple waveforms and envelope features could be more beneficial for the classification of seizure periods and/or stages than that of each individual. To this end, three features extracted from the two contact pairs with the clearest differences in these features across periods/stages determined by the consensus of all viewers (i.e., C4-P4 and Cz-Pz for scheme 1, C4-P4 and C3-P3 for scheme 2, and C3-P3 and Fp2-F8 for scheme 3), referring to Figs. 3, 5, and 6, were selected and summed across subjects. Figure 9 shows scatter plots for the three features (complex envelope, epileptic sharpness, and DoN). Figure 9A shows a cluster of red points (i.e., seizure period) with a cross-section that differentiates the seizure periods from the nonseizure periods. The seizure periods presented a higher envelope power and epileptic sharpness with an elevated DoN. To further investigate the discriminability around the onset of a seizure event, Fig. 9B presents a cluster of magenta points (i.e., stage 1) with a cross-section that differentiates the start stage from nonseizure periods. The start stage showed a higher epileptic sharpness and envelope power with a tendency for DoN. For the termination of a seizure event, Fig. 9C demonstrates two clusters of blue points (i.e., stage 3) with cross-sections that differentiate the end stages from the start stages. The end stage presented higher DoN in general. The sharper cluster corresponded to a lower DoN, and the less sharp cluster had a higher DoN.

## Discussion

This work seeks to establish a set of methods to investigate the evolution of neonatal epileptic patterns. Previous studies have primarily focused on amplitude-based measures [34] or spectral analyses [35] to differentiate epileptic from normal brain states. However, nonlinear characteristics [36] and increased rhythmicity [37] during seizures contribute to the highly complex background of neonatal EEG signals [17]. Figure 1A demonstrates the multichannel time series of one EEG set, starting with several intermittent bursts, followed by consecutive and strong neural firing. While the temporal complex envelope captures some morphological changes, comprising rhythmic delta rhythms, spikes, sharp waves, and slow complexes within a seizure event (Fig. 1A), it may not fully reflect refined neonatal seizure dynamics. Therefore, two waveform quantifying methods (i.e., epileptic sharpness and DoN) were introduced innovatively in neonatal seizure detection. These features, which capture subtle morphological changes, hold significant relevance in depicting the intricate dynamics of neonatal seizure activity. These novel methodologies not only expand the scope of neonatal epileptic signal analysis but also hold the potential to revolutionize how we approach real-time monitoring in neonatal care.

The ANOVA results shown in Table S1 suggest that all three features could significantly differentiate a seizure period from a nonseizure period. Notably, DoN is the only feature with no significant interaction between the seizure period and the number of viewers, indicating its insensitivity to viewers’ judgment. Similarly, the complex envelope was generally higher during the seizure periods. On the other hand, a significant interaction was shown for sharpness, and the difference was especially prominent only when all experts identified a seizure. Overall, the occurrence of a seizure event comes with stronger envelope power, sharper patterns, and increased nonlinearities. Our results suggest the potential of all features as robust biomarkers for identifying and characterizing seizure events.

We also performed classic epileptic feature analyses, such as band power, ER, and RMS, which showed that delta, alpha, beta, gamma power, ER, and RMS significantly differentiate seizure periods from nonseizure periods. These findings align with previous works indicating variations in band power [38,39]. Concerning (a) the seizures resulting from the temporary disruptions of the normal brain state caused by collective and excessive neuronal activities, (b) the complex envelope as an integration of spectral power across multiple frequency bands, plus (c) the efficient and real-time computation for the complex envelope without constraint to a window function, all support the complex envelope as an ideal feature that can be used to characterize neonatal seizure dynamics.

To address the challenge of differentiating finer segmentation within a seizure event, we investigated the temporal evolution of DoN and complex envelope. Our results reveal an increased nonlinearity toward the end of an undisputed seizure period. In contrast, the complex envelope reaches a maximum in the middle of the seizure period, indicating its association with the occurrence of an undisputed seizure event. These observations highlight the different roles of these temporal features in characterizing neonatal seizure dynamics. Furthermore, conventional metrics like ER and RMS demonstrate limitations in capturing the nuances of seizure dynamics. ER can only identify epileptic seizures and cannot reflect their progression. While RMS offers consistent information about finer segmentation within a seizure event, it lacks the comprehensive insights provided by complex envelope power, making it less suitable for this specific application. Also, the optimized cross-sections for clustering in Fig. 9 involve the main contributions from at least two of the three features, supporting their complementarity in monitoring the evolution of neonatal seizures.

The ROC curve was applied to evaluate the performance of classification schemes by which subjects were classified. The epileptic sharpness demonstrates the best discriminability in classifying the seizure from the nonseizure periods, with envelope power following in second place. For the identification of seizure onset, the performance of the envelope power outperforms, closely followed by sharpness. On the other hand, for the identification of the shift to terminate a seizure, DoN has the best performance. These findings support the development of automated methods for neonatal seizure detection and monitoring.

Evidence has shown that synchronization in rhythmic inputs may sharpen oscillatory activities [28,30,40] and impact brain wave network interactions (cross-frequency coupling, time delay stability, etc.) [41–45], while desynchronization may occur with the progression of seizures [46,47]. Our results support the possible linkages between seizure events and synchronized neural activities compared with the nonseizure periods. On the other hand, the elevated DoN toward the end stage within a seizure event implies that the epileptic oscillation becomes less sinusoidal as time evolves, along with a decrease in envelope power. One possible contributing factor to the elevated DoN may be the extreme misalignment between multifrequency rhythms merged with the epileptic oscillation of interest toward the end of a seizure event. That is, the increased phase slips derive instantaneous frequencies. Specifically, the progressive decrease in the entrainment to the epileptic oscillation of interest plus the involvement of the competing oscillations results in a relatively pure tone transition into a more irregular pattern, thus facilitating the termination of an epileptic event. Such change may come with decreased envelope power.

Additionally, distinct topography distributions, in the complex envelope, sharpness, and DoN, were observed by an evolving neonatal seizure, guiding feature extractions in relevant montages. The complex envelope is highest around Cz-Pz, sharpness peaks are highest around C3 and C4, and DoN is highest in the frontal lobe. Moreover, topography distributions shift across seizure stages, with envelope power showing its highest relative difference in the mid-stage, sharpness revealing fewer differences in the end stage, and DoN exhibiting a clear relative difference around the frontal area at the end stage.

Furthermore, compared to the existing algorithms [46] that require approximately 30 ms per segment in computational time, our proposed method significantly reduces the cost (Table S6). This substantial reduction in computational time empowers the detection of seizures over multiple dimensions in temporal dynamics, which facilitates real-time seizure detection on the condition of wearable devices with limited resources. Our approach will have the potential to develop an epileptic activity monitor and personalized clinical decision-making tool [47].

One limitation of this work could be that the subtypes of neonates with suspicious seizure events were not included; thus, whether our observations would be accurate for various subtypes of neonatal seizure remains to be validated. The lack of comprehensive annotation with seizure zones or the diversity of seizure zones resulted in the difficulty of correlating them with specific spatial regions of characteristics. On the other hand, each seizure event was clinically annotated as a fixed 1-s epoch; thus, the progression of finer seizure dynamics requires further exploration. The last limitation is that the aim of the present study centered around the effectiveness of temporal features in predicting seizure events; thus, involving features derived from the spectral band power as a whole in prediction is beyond our scope.

## Data Availability

Data of this paper are available by emailing chien-hung.yeh@bit.edu.cn.
