# Supplementary material for: Quantifying Sharpness and Nonlinearity in Neonatal Seizure Dynamics
Source: Cyborg Bionic Syst. 2024 Jan 25;5:0076. doi: 10.34133/cbsystems.0076 (PMC10809840; doi:10.34133/cbsystems.0076)
Supplement: Supplementary 1 — Figs. S1 to S5 Tables S1 to S6 [file cbsystems.0076.f1.docx]

**Fig. S1** Comparisons of periods (seizure and nonseizure) using band power include (a) delta, (b) theta, (c) alpha, (d) beta, and (e) gamma bands. All comparisons are grouped by the number of the agreed-upon viewers (1, 2, and 3). ** and *** stand for p values < 0.001 and 0.0001, respectively.

**Fig. S2** Comparisons of periods (seizure and nonseizure) using features include (a) ER and (b) RMS. All comparisons are grouped by the number of the agreed-upon viewers (1, 2, and 3). ** and *** stand for p values < 0.001 and 0.0001, respectively.

**Fig. S3** Comparisons of stages (starting, middle, and end seizure stages), grouped by the number of the agreed viewers (1, 2, and 3), using neonatal epileptic EEG characteristics including (a) ER and (b) RMS. * and *** stand for p value < 0.05 and 0.0001, respectively.

**Fig. S4** Comparisons of periods (seizure and nonseizure) using features from CHB-MIT dataset including (a) the complex envelope, (b) epileptic sharpness, (c) DoN, (d) ER, (e) RMS, as well as (f) delta, (g) theta, (h) alpha, (i) beta, and (j) gamma bands power. ** and *** stand for p values < 0.001 and 0.0001, respectively.

**Fig. S5** Comparisons of stages (starting, middle, and end seizure stages) using features from CHB-MIT dataset including (a) complex envelope, (b) DoN, (c) ER, and (d) RMS. * and *** stand for p value < 0.05 and 0.0001, respectively.

Table S1

Comparisons of periods, contacts, and viewers using EEG temporal characteristics.

|  |  | **Complex Envelope** | | | **Sharpness** | | | **DoN** | |
| --- | --- | --- | --- | --- | --- | --- | --- | --- | --- |
| **Factors** | **DF** | **F Value** | **P Value** | **F Value** | | **P Value** | **F Value** | | **P Value** |
| Period | 1 | 621.17 | **0.0000** | 38.62 | | **0.0000** | 26.18 | | **0.0000** |
| Contact | 17 | 4.27 | **0.0000** | 62.46 | | **0.0000** | 21.46 | | **0.0000** |
| Viewer | 2 | 115.07 | **0.0000** | 4.73 | | **0.0089** | 0.61 | | 0.5414 |
| Period*Contact | 17 | 1.38 | 0.1358 | 2.62 | | **0.0003** | 1.78 | | **0.0249** |
| Period*Viewer | 2 | 141.93 | **0.0000** | 9.08 | | **0.0001** | 0.34 | | 0.7152 |
| Contact*Viewer | 34 | 0.50 | 0.9936 | 0.40 | | 0.9992 | 0.27 | | 1.0000 |

Neonatal epileptic EEG characteristics include complex envelope, epileptic sharpness, and DoN. Factors include periods (seizure and nonseizure), contacts (18 bipolar contact pairs), the number of the agreed-upon viewers (1, 2, and 3), and their pairwise interactions. Repeated ANOVA analysis was used. Bold fonts indicate *p* values < 0.05.

Table S2

Comparisons of periods and contacts using EEG temporal characteristics grouped by viewers' number.

|  |  |  | **Complex Envelope** | | **Sharpness** | |
| --- | --- | --- | --- | --- | --- | --- |
| **V*♯*** | **Factors** | **DF** | **F Value** | **P Value** | **F Value** | **P Value** |
| 1 | Period | 1 | 46.11 | **0.0000** | 4.53 | **0.0336** |
|  | Contact | 17 | 1.79 | **0.0241** | 23.59 | **0.0000** |
|  | Period*Contact | 17 | 0.22 | 0.9996 | 0.75 | 0.7548 |
| 2 | Period | 1 | 134.44 | **0.0000** | 0.97 | 0.3240 |
|  | Contact | 17 | 2.43 | **0.0009** | 19.18 | **0.0000** |
|  | Period*Contact | 17 | 0.63 | 0.8735 | 1.28 | 0.1997 |
| 3 | Period | 1 | 463.42 | **0.0000** | 54.07 | **0.0000** |
|  | Contact | 17 | 1.45 | 0.1060 | 21.06 | **0.0000** |
|  | Period*Contact | 17 | 1.12 | 0.3318 | 0.87 | 0.6058 |

Neonatal epileptic EEG characteristics include complex envelope and epileptic sharpness. The number of the agreed-upon viewers includes 1, 2, and 3. Factors include periods (seizure and nonseizure), contacts (18 bipolar contact pairs), and their interactions. Repeated ANOVA analysis was used. Bold fonts indicate *p values* < 0.05.

Table S3

Comparisons of periods and viewers using EEG temporal characteristics grouped by contacts.

| **Sharpness** | | | | | | |
| --- | --- | --- | --- | --- | --- | --- |
|  | **Fp2-F4** | **F4-C4** | **C4-P4** | **P4-O2** | **Fp1-F3** | **F3-C3** |
| Period | 0.6938 | **0.0005** | **0.0000** | **0.0000** | **0.0495** | **0.0000** |
| Viewer | 0.3839 | 0.0729 | **0.0308** | **0.0172** | 0.9353 | 0.6222 |
| Period*Viewer | 0.1029 | **0.0206** | **0.0122** | **0.0033** | 0.3751 | 0.3567 |
|  | **C3-P3** | **P3-O1** | **Fp2-F8** | **F8-T4** | **T4-T6** | **T6-O2** |
| Period | **0.0000** | **0.0004** | 0.1507 | 0.8194 | **0.0274** | **0.0058** |
| Viewer | 0.1259 | **0.0158** | 0.1258 | 0.2496 | 0.3918 | 0.9570 |
| Period*Viewer | **0.0107** | **0.0074** | 0.0507 | 0.1080 | 0.1129 | 0.4352 |
|  | **Fp1-F7** | **F7-T3** | **T3-T5** | **T5-O1** | **Fz-Cz** | **Cz-Pz** |
| Period | **0.0237** | **0.0320** | **0.0305** | **0.0187** | **0.0011** | **0.0000** |
| Viewer | 0.8288 | 0.6796 | 0.3152 | **0.0278** | 0.2387 | 0.1587 |
| Period*Viewer | 0.3163 | 0.3176 | 0.1732 | **0.0103** | **0.0740** | **0.0102** |
| **DoN** | | | | | | |
|  | **Fp2-F4** | **F4-C4** | **C4-P4** | **P4-O2** | **Fp1-F3** | **F3-C3** |
| Period | 0.4046 | 0.6389 | 0.9848 | 0.0965 | 0.2572 | 0.6104 |
| Viewer | 0.3945 | 0.7088 | 0.9007 | 0.8142 | 0.2656 | 0.8268 |
| Period*Viewer | 0.4516 | 0.7421 | 0.9402 | 0.8969 | 0.2828 | 0.8281 |
|  | **C3-P3** | **P3-O1** | **Fp2-F8** | **F8-T4** | **T4-T6** | **T6-O2** |
| Period | 0.1181 | **0.0095** | **0.0452** | **0.0000** | **0.0196** | 0.1452 |
| Viewer | 0.6904 | 0.6595 | 0.7279 | 0.9921 | 0.5209 | 0.8520 |
| Period*Viewer | 0.6706 | 0.8284 | 0.6842 | 0.9803 | 0.6029 | 0.5109 |
|  | **Fp1-F7** | **F7-T3** | **T3-T5** | **T5-O1** | **Fz-Cz** | **Cz-Pz** |
| Period | **0.0240** | **0.0205** | **0.0500** | 0.0736 | 0.2095 | 0.2211 |
| Viewer | 0.7720 | 0.5072 | 0.5660 | 0.9383 | 0.6926 | 0.9228 |
| Period*Viewer | 0.6798 | 0.5610 | 0.6046 | 0.9108 | 0.7960 | 0.8518 |

Neonatal epileptic EEG characteristics include epileptic sharpness and DoN. The contacts include 18 bipolar contact pairs. Factors include periods (seizure and nonseizure), the number of the agreed-upon viewers (1, 2, and 3), and their interactions. Repeated ANOVA was used. Bold fonts indicate *p* values < 0.05.

Table S4

Comparisons of stages, contacts, and viewers using EEG temporal characteristics.

|  |  | **Complex Envelope** | | **Sharpness** | | **DoN** | |
| --- | --- | --- | --- | --- | --- | --- | --- |
| **Factors** | **DF** | **F Value** | **P Value** | **F Value** | **P Value** | **F Value** | **P Value** |
| Stage | 2 | 22.36 | **0.0000** | 0.15 | 0.8577 | 0.05 | 0.9510 |
| Contact | 17 | 10.11 | **0.0000** | 82.15 | **0.0000** | 20.47 | **0.0000** |
| Viewer | 2 | 496.31 | **0.0000** | 32.12 | **0.0000** | 3.30 | **0.0370** |
| Stage*Contact | 34 | 0.15 | 1.0000 | 0.04 | 1.0000 | 0.23 | 1.0000 |
| Stage*Viewer | 4 | 15.47 | **0.0000** | 0.76 | 0.5493 | 2.68 | **0.0299** |
| Contact*Viewer | 34 | 1.77 | **0.0038** | 1.04 | 0.4057 | 0.82 | 0.7544 |

Neonatal epileptic EEG characteristics include complex envelope, epileptic sharpness, and DoN. Factors include stages (starting, middle, and end seizure stages), contacts (18 bipolar contact pairs), the number of the agreed-upon viewers (1, 2, and 3), and their pairwise interactions. Repeated ANOVA was used. Bold fonts indicate *p* values < 0.05.

Table S5

Comparisons of stages and contacts using EEG temporal characteristics grouped by viewers' number.

|  |  |  | **Complex Envelope** | | **DoN** | |
| --- | --- | --- | --- | --- | --- | --- |
| **V*♯*** | **Factors** | **DF** | **F Value** | **P Value** | **F Value** | **P Value** |
| 1 | Stage | 2 | 0.93 | 0.3942 | 0.44 | 0.6419 |
|  | Contact | 17 | 3.29 | **0.0000** | 9.02 | **0.0000** |
|  | Stage*Contact | 34 | 0.10 | 1.0000 | 0.25 | 1.0000 |
| 2 | Stage | 2 | 4.45 | **0.0118** | 1.17 | 0.3104 |
|  | Contact | 17 | 5.86 | **0.0000** | 7.09 | **0.0000** |
|  | Stage*Contact | 34 | 0.10 | 1.0000 | 0.34 | 0.9999 |
| 3 | Stage | 2 | 42.57 | **0.0000** | 6.11 | **0.0023** |
|  | Contact | 17 | 6.91 | **0.0000** | 8.41 | **0.0000** |
|  | Stage*Contact | 34 | 0.22 | 1.0000 | 0.35 | 0.9998 |

Neonatal epileptic EEG characteristics include complex envelope and DoN. The number of the agreed viewers includes 1, 2, and 3. Factors include stages (starting, middle, and end seizure stages), contacts (18 bipolar contact pairs), and their interactions. Repeated ANOVA was used. Bold fonts indicate *p* values < 0.05.
